# The Role of Interventional Endoscopic Ultrasound in Liver Diseases: What Have We Learnt?

**DOI:** 10.1155/2021/9948979

**Published:** 2021-06-28

**Authors:** Cosmas Rinaldi A. Lesmana, Maria Satya Paramitha, Rino A. Gani

**Affiliations:** ^1^Department of Internal Medicine, Hepatobiliary Division, Dr. Cipto Mangunkusumo National General Hospital, Universitas Indonesia, Jakarta, Indonesia; ^2^Digestive Disease and GI Oncology Center, Medistra Hospital, Jakarta, Indonesia

## Abstract

Chronic liver disease (CLD) is still a major problem, where the disease progression will lead to liver cirrhosis (LC) or hepatocellular carcinoma (HCC). Portal hypertension (PH) management and loco-regional therapy for HCC have become the cornerstones in advanced liver disease management. Recently, there are studies looking at the potential role of interventional endoscopic ultrasound (EUS) in liver diseases. EUS may be useful in vascular changes of the digestive wall evaluation, performing dynamic assessment of hemodynamic changes, predicting variceal bleeding and rebleeding risk, and assessing the pharmacological effects. In PH management, EUS-guided vascular therapy—which revolves around glue injection, endovascular coil placement/embolization, and combination of both—has shown promising results. As a diagnostic modality for liver cancer, the implementation of EUS in liver diseases is currently not only limited to liver biopsy (EUS-LB) but also in shear-wave elastography (SWE) and portal pressure gradient measurement, as well as portal vein sampling. The application of EUS-guided radiofrequency ablation (EUS-RFA) and tumor injection can also overcome the limitations shown by both modalities without EUS. Nevertheless, establishing EUS as a firm diagnostic and therapeutic modality is still challenging since the performance of interventional EUS requires high expertise and adequate facilities.

## 1. Introduction

Since the introduction of endoscopic ultrasound (EUS) in the 1980s, innovation in diagnostic as well as therapeutic EUS-guided has emerged, especially for pancreatobiliary disorders [1, 2]. Chronic liver disease (CLD) is a worldwide major problem, which leads to complications, such as liver cirrhosis (LC) or hepatocellular carcinoma (HCC). The diagnosis and management of portal hypertension (PH) have become the cornerstones in daily practice. Recently, there have been studies looking about the role of interventional EUS in liver disease. However, several factors, such as cost issue, availability, and high expertise to perform this procedure are still become debatable conditions [1, 3]. This review will discuss about the potential role of EUS from various clinical evidence in liver disorders—including PH and HCC—as two major causes of morbidity and mortality.

## 2. The Diagnostic Role of Endoscopic Ultrasound

### 2.1. The Role of Endoscopic Ultrasound in Liver Disease Evaluation

Liver biopsy is still considered as the gold standard for establishing the unknown abnormality of liver enzymes or for determining the stages of liver disease, especially in early liver cirrhosis detection. Conventional liver biopsy method, percutaneous approach, is still the simplest way to get liver specimen in daily practice. However, several major issues have been raised regarding this method, such as severe referred pain to the right shoulder, bleeding complication, bile duct injury, sampling error, and uncooperative patient. Another way to obtain appropriate liver tissues is through trans-jugular liver biopsy (TJLB). In patients with ascites, coagulopathy, and other conditions in which portal pressure needs to be measured, this procedure seems to be the ideal way. However, its invasiveness, requirement of skillful and experienced operator, and the risk of radiation exposure are the main things to be counted in real everyday practice [4, 5]. Based on the pathologist's point of view, the length of the tissue sample, portal tract, and bile duct are the most important things for establishing the diagnosis. Another major drawback, the liver condition, such as fatty liver, which is usually characterized by the brightness of the liver is sometimes not easy to detect early changes of liver parenchymal injury as well as possibility of small liver lesion development [6].

As a diagnostic modality, the liver segments are recognized by EUS as follows: branches of portal vein with thick and hyperechoic walls (Doppler positive), branches of hepatic veins with thin and nonreflective walls (Doppler positive), biliary radicals with hyperechoic walls (Doppler negative), venosum and teres ligaments with thick and hyperechoic structures, and other structures, such as gallbladder, falciform ligament, and liver hilum. Anatomically, liver is supplied by portal vein and hepatic artery; thus, the application of ultrasound contrast can show three vascular phases: the arterial phase (starts within 20 seconds after the injection and continues for roughly 30–45 seconds); the portal venous phase (ends in approximately 2 minutes); and the late phase (persists until the contrast agent can be cleared from the circulation; which usually takes around 6 minutes). The technical mechanism, in which, the EUS transducer can be placed close to the liver without being affected by interposing structures, is also one of the advantages of EUS over transabdominal ultrasound, especially also in obese patients [7]. Study by Shuja et al. [8], on the comparison between EUS-guided liver biopsy and percutaneous approach as well as transjugular liver biopsy, showed that even though the mean of complete portal triad was found more in the imaging-guided percutaneous approach compared to EUS-guided approach (13.6 vs. 10.8, *p* ≤ 0.01); however, total complications' rate was found more in the percutaneous approach compared to EUS-guided approach. In the EUS-guided approach group, there were no significant complications. Another retrospective study on liver biopsy methods comparison between percutaneous approach and EUS-guided approach by Ali et al. [6] showed that the median number of portal tracts was found higher in percutaneous group compared to EUS-guided group, but in EUS-guided group have significant in shorter hospital stay (*p* ≤ 0.004), and less pain complication when compared to percutaneous group (*p* ≤ 0.0009) [8].

### 2.2. The Role of Endoscopic Ultrasound in Portal Hypertension Evaluation

Normally, portal vein has a low pressure (≤5 mmHg) and a low-resistance system; acting as the major outflow tract for splanchnic circulation. However, increased in portal pressure can be due to by prehepatic, intrahepatic, and posthepatic events [9].

A clinically significant portal hypertension (CSPH) is defined as a porto-hepatic gradient ≥10 mmHg. Different levels of risk are correlated with the degree of portal hypertension. Portal hypertension affects the natural history and prognosis of cirrhosis by causing hyperdynamic circulatory syndrome, which may result in esophageal varices (EV), gastrointestinal bleeding, spontaneous bacterial peritonitis, hepatorenal syndrome, hepatopulmonary syndrome, and/or hepatic encephalopathy [5, 6]. Variceal bleeding is correlated with HVPG higher than 12 mmHg, while the risk of hepatocellular carcinoma increased when HVPG is greater than 10 mmHg. A systematic review involving 23,797 subjects from 118 studies also demonstrated portal hypertension as one of the main predictors of worse prognosis in cirrhotic patients [9, 10].

EUS-Doppler, especially, is more advantageous in evaluating vascular changes in patients with portal hypertension, compared to endoscopy since EUS-Doppler can detect gastroesophageal varices along with periesophageal collateral veins, paraesophageal collateral veins, and perforating veins. EUS-Doppler can also evaluate parameters for bleeding; for instance, the size and thickness, as well as hemodynamic changes of portal veins, azygos veins, and left gastric vessels [7].

Another potential clinical application of EUS in portal hypertension, according to previous studies, may include evaluating vascular changes of the digestive wall, performing dynamic assessment of hemodynamic changes with EUS-guided portal vein catheterization, predicting variceal bleeding and rebleeding risk, and assessing pharmacological effects [7]. In comparison to upper endoscopy, EUS-Doppler has been indicated as a better modality to detect esophageal and gastric varices. A study by Burtin et al. has shown that the higher the endoscopic grade of esophageal varices, the higher the sensitivity of EUS [11]. For small esophageal varices, the detection capability can be increased by utilizing small water-filled balloons, small 20 Hz ultrasound transducers, high-frequency ultrasound miniature probes, and high-resolution endoluminal sonography [7]. EUS-guided portal vein catheterization can also overcome the transjugular approach. A prospective case series has demonstrated high technical success rate without any adverse events in portal pressure gradient measurement using a 7.5 MHz linear echoendoscope and a 19 G FNA needle [12, 13].

Performing EUS-Doppler-guided manometry to indirectly measure intravariceal pressure has also been demonstrated as a promising method in evaluating variceal bleeding risk. Other predictors, for instance, hematocystic spots on the surface of esophageal varices, high blood flow variceal velocities, thin gastric variceal walls, and size and number of paraesophageal and periesophageal collateral veins, are also shown to be associated with bleeding risks [7]. EUS can also predict future variceal bleeding by calculating each 1 cm^2^ increase of variceal cross-sectional surface area, which is equal to 76-fold increase annual risk of variceal bleeding with high sensitivity and specificity (83% and 75%, respectively) [14]. High diagnostic yield (sensitivity 89.2%, specificity 90.5%, and AUC 0.946) has also been demonstrated by EUS in predicting rebleeding of esophageal varices following endoscopic variceal ligation [15]. Diameter of paraesophageal varices was shown by another study to be a better predictor of rebleeding risk since it exhibited higher sensitivity (70.6%) and higher area under the receiver operating characteristics curve (0.801) [16]. Furthermore, combination of EUS morphological assessment of varices and portal pressure measurement can be an objective tool to stratify the risk of rupture, as well as evaluating the effect of vasoactive agents' injection on the superior portosystemic collateral circulation and portal venous flow of patients with portal hypertension [7].

Another application of interventional EUS in diagnostic confirmation is through EUS-guided portal vein sampling. Aside from circulating tumor cells (CTCs), other cancer-derived products, such as circulating tumor DNAs and exosome, are being studied as minimally invasive tools for assessment of solid tumor characteristics and/or distant metastases. Nevertheless, the approach of assessing CTCs from peripheral blood samples still displays several limitations since CTCs are often shed into the bloodstream in the form of single cells (20–30 um) or micro emboli, which may affect the quality of the quantification [17]. A prognostic significance of counting CTCs from portal vein has been shown in a multivariate analysis by Tien et al. Portal CTCs appeared to have high specificity (95.4%) in predicting liver metastases within six months after surgery [18]. There are currently two methods in acquiring samples from portal veins: transhepatic routes to access the transgastric or transduodenal intrahepatic portal vein or subsidiary branch or transduodenal extrahepatic route. It is also important to perform Doppler evaluation of the liver prior to launching the needle into portal vein, in order to confirm the patency of hepatic artery, hepatic veins, and portal vein. Despite being a potential advancement as EUS-guided diagnostic intervention, bleeding and blood sample clotting still become a significant troubleshooting in this procedure. It is, therefore, recommended to maintain the international normalized ratio (INR) of the patient to be less than 1.5 and the platelet count to be more than 50 × 10^9^/L to minimize the risk of immediate or delayed gastrointestinal hemorrhage. Administration of sclerosants, cyanoacrylate, thrombin, and coils to the site of portal entry may also decrease the likelihood of bleeding events, although, further evidences are still needed. The possibility of blood sample clotting also contributes in lowering the accuracy of the sampling. Consequently, to lower the risk of blood clotting in the blood sample, the sample needs to be instantly transferred into vacutainer tube, which contains cell preservatives, from the negative suction syringe. Priming the syringe of needle with a small amount of anticoagulant solution may also reduce the possibility of blood clotting [17].

### 2.3. The Role of Endoscopic Ultrasound in Liver Lesion and Liver Malignancy

Nowadays, primary liver cancer (hepatocellular carcinoma/HCC) still becomes a prominent healthcare burden in Western as well as Asian countries. As the fourth most common cause of cancer-related death worldwide, it is estimated that more than one million patients will die due to liver cancer in the next ten years [19, 20]. The five-year survival rate of liver cancer is approximately 18%, with the most common histologic subtype is hepatocellular carcinoma (HCC), mostly due to chronic viral infection, alcohol abuse, fatty liver, or other underlying liver diseases [21]. The second highest incidence rates of HCC were found in Southeast Asia, with an age-standardized rate (ASR) of 22.2 per 100,000 in males and 7.2 per 100,000 in females. Chronic hepatitis B virus (HBV) and hepatitis C virus (HCV) infections are still considered as the most common risk factors for HCC (60%) worldwide. As one of the HBV endemic areas, the incidence rates of HCC were quite high (13.4 per 100,000 in males and 4.0 per 100,000 in females) [22]. A recent retrospective study conducted in the biggest national tertiary referral hospital in Indonesia (the biggest country in Southeast Asia), still demonstrated poor survival of HCC patients. This happened because, in spite of the availability of advanced treatment modalities, many patients still came in the late stages of the disease [23].

The indications of EUS in detecting focal liver lesion have been expanded as the landscape of liver disease has also been changing rapidly. The implementation of EUS in liver diseases is currently not limited only on focal liver lesion biopsy but also in shear-wave elastography (SWE) innovation [24]. The high diagnostic yield of EUS in detecting lesions sized less than 1 cm also contributes to EUS being considered as a complementary modality to computed tomography (CT) and magnetic resonance imaging (MRI). The use of contrast-harmonic EUS (CH-EUS) and EUS-elastography (EUS-E) has been proposed to increase the diagnostic accuracy of EUS since the intrinsic ability of EUS is limited to examining only the left lobe and small portion of the right lobe [25].

Aside from high diagnostic yield, EUS-LB has also demonstrated fewer adverse events compared to traditional percutaneous approaches. These advantages may be contributed by real-time ultrasound guidance, rapid recovery time, the possibility of producing several needle actuations within the liver with only a single puncture through the liver capsule [24]. Moreover, it has been estimated that the potential sampling errors of single-blind percutaneous liver biopsy for detection of cirrhosis may occur in up to 30% cases [26].

As has been mentioned before, contrast-enhancement EUS (CE-EUS) has emerged as a more appropriate method to diagnose focal liver lesions; either through CE-EUS with Doppler method (CE-EUS-D) or CH-EUS. Generally, CE-EUS-D is better in differentiating vascular-rich and hypovascular areas of target lesion, while CH-EUS is able to give more detailed vasculature images of target lesion. Today, the most widely used ultrasound contrast agents are SonoVue and Sonazoid, produced from microbubbles with a shell of phospholipids and sulfur hexafluoride gas, allowing visualization of small vessels in the capillary bed, resulting into more dynamic depiction of capillary microvascularization. Compared to CT and MRI, CE-EUS shows a real-time performance, prolonged enhancement of vascular system due to confinement in the vascular space without extravasation into the interstitial space and higher resolution. CE-EUS has also shown a better tolerance and safety profile, especially in patients with renal insufficiency [7, 27]. Moreover, CE-EUS has also shown higher diagnostic accuracy, compared to contrast-enhanced CT, in detecting residual tumors after TACE (96.2% vs. 77.7%) [28].

In addition, the utilization of EUS with real-time elastography has been deemed to be more auspicious compared to transabdominal FibroScan in detection of liver cirrhosis, fibrosis, and steatosis. A retrospective study by Sandulescu et al., in differentiating benign and malignant focal liver lesions, demonstrated a promising diagnostic performance of EUS-elastography; shown by 92.5% sensitivity, 88.8% specificity, 88.6% accuracy, 86.7% positive predictive value, and 92.3% negative predictive value. Further studies, though, are still necessary due to the small sample size of the study [7, 29].

## 3. The Therapeutic Role of Endoscopic Ultrasound

### 3.1. The Role of Endoscopic Ultrasound in Portal Hypertension: Vascular Intervention

Management of portal hypertension varies according to the complications found in clinical practice. Around 60% of patients with decompensated cirrhosis are present with EV; thus, esophagogastroduodenoscopy (EGD) is still recommended as a primary prophylaxis screening tool of subsequent hemorrhage [9]. In patients with large varices, definitive endoscopic treatment (endoscopic variceal band ligation) and the use of vasoactive agents (i.e., vasopressin analogue and somatostatin analogue) has given significant benefit in decreasing bleeding-related mortality [30, 31].

Assistance of EUS in management of portal hypertension has been reported in many studies. The first pilot study of applying EUS-Doppler in the esophageal variceal ligation technique was first conducted by Nagamine et al. The study, specifically, underlined the advantage of EUS in better identification of variceal zones and good intermediate-term outcome during study follow-up [32]. Previous evidence also showed the potency of EUS-guided sclerotherapy in the management of esophageal varices; especially in reducing the risk of recurrence and diameter of azygos vein [33, 34]. A randomized controlled trial demonstrated lower recurrence rate in EUS-guided sclerotherapy compared to endoscopic sclerotherapy (4 patients vs. 2 patients). This trial also showed that persisting esophageal collateral vessels after sclerotherapy was a statistically significant risk factor for recurrence (*p*=0.003) [33].

Decompression of portal system by creating a low-resistance channel between portal vein and inferior vena cava is usually achieved by Transjugular Intrahepatic Portosystemic Shunt (TIPSS) procedure [26]. This procedure, however, can be challenging in the case of obstructed inferior vena cava and/or hepatic veins. Catheter manipulation through intra-thoracic inferior vena cava and right atrium can also cause life-threatening adverse events; especially in patients with history of cardiopulmonary abnormalities [7]. The application of EUS-guided TIPSS, therefore, is considered to be potentially beneficial since it does not need to be advanced through intrathoracic inferior vena cava. This procedure also has lower radiation exposure during deployment of LAMS in animal models [35, 36].

At this moment, the role of EUS-guided interventions in vascular therapy has revolved around glue injection, endovascular coil placement/embolization, and combination of both. Several advantages offered by EUS-guided vascular therapy include the use of Doppler ultrasound, which allows better ability to distinguish varices from gastric folds or other lesions: as well as providing easier access for direct injection into the varix [24].

Throughout the years, cyanoacrylate glue injection has become a pillar in the management of acute bleeding, as well as secondary prevention. N-2-Butyl-cyanoacrylate is a watery liquid, which will turn into solid state if it is added into a medium containing hydroxyl ion. Injection of this substance into the varix will lead to immediate polymerization of glue because of the hydroxyl ions in blood. The glue polymerization will form a harder substance which causes obturation of the lumen of varix; thus, rapidly establishing hemostasis and preventing rebleeding at the same time. The application of EUS-guided glue injection provides higher precision of injection into the varix and enables real-time visualization at the same time, which may decrease the risk of embolization [24]. An open-basis case-series study performed in gastric varices patients showed successful results in EUS-guided injection of cyanoacrylate-lipiodol at the level of perforating veins without recurrent bleeding or other major complications [37]. Common complications of cyanoacrylate glue are the formation of giant ulcer at the site of injection, bleeding and/or rebleeding, as well as systemic complications, such as pyrexia and mild abdominal pain. Factors which may influence the occurrence of complications can be classified into patient-related factors and technique-related factors. Large gastric varices with draining gastrorenal, gastrocaval, or gastrolienorenal shunts are the most common patient-related factors for complications. Overdilution of cyanoacrylate with lipiodol is commonly found as the technical error since overdilution may cause prolonged polymerization and, thereupon, increasing the risk of embolization [38].

Another method of EUS-guided vascular therapy is by placing microcoils with synthetic fiber that can promote clot formation, leading to embolization through obliteration of varices [24]. This technique has been widely performed as monotherapy and combination therapy. A case report of refractory ectopic variceal bleeding demonstrated successful hemorrhage control with only minimal self-limited bleeding on the puncture location after EUS-guided coil embolization procedure [39]. A 6-year retrospective study on EUS-guided injection of coils and cyanoacrylate therapy on gastric fundal varices demonstrated high efficacy of this procedure; indicated from more than 99% technical success among 151 patients with low rebleeding rate (3%) [40]. These findings were supported with similar evidence in a case series with EUS-guided injection of coils and cyanoacrylate in gastric fundal varices cases with abnormal shunts. No adverse events were reported from the cases. Combining coils and cyanoacrylate glue can be more effective because the coils may act as a “scaffold” within blood vessels; allowing cyanoacrylate glue to be better incorporated to the varices [41]. On the other hand, in a comparison multicenter study between EUS-guided coil and cyanoacrylate therapy for gastric varices, it was exhibited that EUS-guided cyanoacrylate therapy resulted into slightly higher varices obliteration rate (94.7% vs. 90.9%) but with higher rate of adverse events (57.9% vs. 9.1%). Further studies with larger sample size are still necessary to validate these findings [42]. A recent randomized trial comparing combination of EUS-guided coil and cyanoacrylate therapy with EUS-guided coil alone showed significantly higher varices obliteration rate in combination group (86.7% and 13.3% in combination group and coil monotherapy, respectively; *p* < 0.001). In addition, combination group also showed higher free-from-reintervention rate compared to coil monotherapy (83.3% vs. 60%) [43]. These findings are further validated with a meta-analysis of three modalities; demonstrating better technical and clinical success in combination therapy compared to cyanoacrylate alone (technical success: 100% vs. 97%, clinical success: 98% vs. 96%; *p* < 0.001) and coil embolization alone (technical success: 99% vs. 97%, clinical success: 96% vs. 90%; *p* < 0.001). Lower adverse event rates were also observed in combination therapy compared to cyanoacrylate alone (10% vs. 21%) [44].

### 3.2. The Role of Endoscopic Ultrasound in Liver Malignancy Management

The therapeutic roles of EUS have been described in many previous studies. One of the most common implementations of therapeutic EUS is for tumor ablation/injection. The most widely used method for HCC and liver metastases, nowadays, is EUS-guided RFA. Throughout the years, several innovations have been proposed to maximize the performance of RFA, for example, by developing a prototype of retractable umbrella-shaped electrode array to produce an effective coagulation necrosis of large areas or by developing a monopolar RFA using thinner and more flexible wire electrode (1 Fr) to gain easier tissue access [45, 46]. A new device combining bipolar RFA and cryotechnology, called cryothermy has also been developed to allow higher efficiency of tissue ablation by establishing cooling cryogenic gas. For other minimally invasive options, neodymium : yttrium-aluminum-garnet (Nd-YAG) laser ablation and high-intensity focused ultrasound (HIFU) have been refined for solid tumors, including HCC and/or liver metastases. Nd-YAG works by directing low-power laser light energy into solid tumor tissues with thinner needles, shorter application time, and the possibility of reusing or resharping the needles. Meanwhile, EUS-HIFU has been mainly put to use in solid tumors closely located to the gastric lumen without gas interposition. HIFU itself has been utilized as a thermal ablation for ablation of liver metastases through surgical or transcutaneous routes [7]. Compared to percutaneous approaches, the application of EUS in RFA is more beneficial, especially in anatomical locations, which are difficult to be visualized and/or reached [47].

The performance of EUS in tumor injection is based on the aim to administer therapeutic agents to target sites with higher precision, in comparison to percutaneous approach. A promising result has been reported by several case series of EUS-guided fine needle ethanol injection in HCC and liver metastases, including in malignant refractory left-sided liver tumors [48, 49]. Another potential palliative therapy is through EUS-guided iodine-125 brachytherapy for left-sided liver tumors, which are not responsive to other transabdominal interventions [48]. Implementation of EUS in chemotherapy injection has also been reported in liver metastases injected with irinotecan-loaded microbeads. In this study, EUS-guided injection was reportedly able to increase the concentrations of irinotecan in the liver with lower systemic exposure; thus, minimizing damage towards nontumor tissues [50].

Experiences of interventional EUS in radiation and embolization therapy have been coming to light in recent years. SBRT, particularly, needs placement of fiducial markers in the target lesion in order to be performed adequately. By utilizing EUS-guided fiducial placement, the photon beams will intersect at a stereotactically determined target; thus, allowing more precise delivery of higher radiation doses. A retrospective study by Choi et al. indicated successful SBRT in 90.6% of patients who previously underwent EUS-guided fiducial placement in patients with pancreatic and hepatic malignancies [7, 51]. On the contrary, the feasibility of inducing hepatic lobar atrophy and hypertrophy of functional liver remnants through EUS-guided portal vein embolization prior to liver resection was first evaluated in an animal study using ethylene-vinyl alcohol copolymer injection (EVAL) into the portal vein [52]. EUS-guided selective intrahepatic portal vein embolization has since emerged as a safe treatment procedure with sufficiently high success rates, both for coil (88.9%) and cyanoacrylate (87.5%) delivery in animal models [53].

## 4. Potential Use of Interventional EUS in Other Liver Conditions (Ascites; Liver Abscess)

Another well-known implementation of EUS-guided procedure is in treating ascites, mainly due to refractory ascites. Generally, paracentesis is a removal procedure of ascitic fluid, using a needle. The first experience of EUS-FNA of pleural and ascitic fluid was reported in 1995. EUS-guided approach is deemed to be potential since it allows easier visualization of ascites. The technical approach is similar to pancreatic cyst drainage, in which an EUS needle is inserted into the fluid collection, and aspiration is conducted due to negative pressure from syringe suction [24, 54]. Traditional EUS-FNA needles (e.g., 25 G and 22 G needles) have been reported to be cost-effective with good success rate [54]. A retrospective study by Suzuki et al. reported the potential use of specialized spring-loaded 22 G FNA needle for EUS-guided paracentesis with 100% success rate and no adverse events [55]. Nonetheless, the risk of infection, which may result into bacterial peritonitis, still becomes one of the potential complications related to EUS-guided paracentesis [24].

The roles of interventional EUS have been indicated, too, in the management of liver cysts and abscess. Symptomatic liver cysts, notably, can be benefitted from EUS-guided aspiration and ethanol lavage therapy since it may replace the needs of surgical treatment or percutaneous aspiration, which were known to have significant morbidity rates [7]. A recent retrospective study also showed the benefit of this through almost 100% reduction of liver cysts within the median 15 months of follow-up [56]. Lower adverse events rate was also found in the use of 1% lauromacrogol as a sclerosing agent compared to ethanol in one case report of giant cyst in the left lobe of the liver [57]. In the management of liver abscess, EUS-guided liver abscess drainage (EUS-AD) has been proposed as an alternative to overcome the limitations of percutaneous liver abscess drainage (i.e., external drainage, patient discomfort due to self-tube removal). A study by Ogura et al. reported significantly shorter hospital stay (21 days in EUS-AD vs. 41 days in percutaneous drainage, *p*=0.03), higher clinical success (100% in EUS-AD vs. 89% in percutaneous drainage), and lower adverse event (0 case in EUS-AD vs. 3 cases in percutaneous drainage) rates in abscesses located in the left liver lobe treated with EUS-AD with fully covered self-expandable metallic stents compared to percutaneous drainage [58]. Another case report also concluded similar success of EUS-AD with SEMS in liver abscess located in the right liver lobe [59]. Recently, a review by Chin et al. from 15 studies demonstrated 97.5% technical success rate and 95% clinical success rate without any major complications (5% complication due to stent migration) of EUS-AD procedure [60].

## 5. Conclusion

In diagnosis and management of liver disorders, exponentially growing evidence has shown potentially significant benefits in the application of EUS; partly due to its real-time imaging of the liver at high resolution. The use of contrast, Doppler ultrasound, and elastography technique can increase the diagnostic yield of EUS. EUS has also established a platform for vascular interventions with high efficacy of combination between EUS-guided cyanoacrylate injection and coil embolization. Moreover, in managing portal hypertension, future studies about EUS-guided creation of intrahepatic portosystemic shunts are still widely opened. In the liver malignancy management, EUS has also been showed to have an important role. To conclude, the main advantage of interventional EUS in liver diseases is the possibility of performing diagnosis and treatment in a single procedure. Nevertheless, firmly establishing EUS as a diagnostic and therapeutic modality is still challenging since the performance of interventional EUS needs high expertise, built from a lot of clinical experiences. The development of interventional EUS is still considered costly and complicated because the adequate facilities are still not distributed evenly in all regions and also the need for highly expert and experience center.

## Data Availability

No data were used to support this study.

## References

[1] Ryozawa S., Fujita N., Irisawa A., Hirooka Y., Mine T. (2017). Current status of interventional endoscopic ultrasound. *Digestive Endoscopy*.

[2] Saraireh H. A., Bilal M., Singh S. (2017). Role of endoscopic ultrasound in liver disease: where do we stand in 2017?. *World Journal of Hepatology*.

[3] Simons-Linares C. R. (2019). From a diagnostic tool to a therapeutic instrument: interventional endoscopic ultrasound. *ACG Case Reports Journal*.

[4] Baron T., Parekh P., Majithia R., Diehl D. (2015). Endoscopic ultrasound-guided liver biopsy. *Endoscopic Ultrasound*.

[5] Johnson K. D., Laoveeravat P., Yee E. U., Perisetti A., Thandassery R. B., Tharian B. (2020). Endoscopic ultrasound guided liver biopsy: recent evidence. *World Journal of Gastrointestinal Endoscopy*.

[6] Nallapeta N., Rao D., Ali A. H., Sharma N., Ibdah J. A., Hammoud G. M. (2020). Endoscopic ultrasound-guided liver biopsy; the pathologist’s perspective. *Journal of Experimental Pathology*.

[7] Campos S., Poley J.-W., van Driel L., Bruno M. J. (2019). The role of EUS in diagnosis and treatment of liver disorders. *Endoscopy International Open*.

[8] Shuja A., Alkhasawneh A., Fialho A. (2019). Comparison of EUS-guided versus percutaneous and transjugular approaches for the performance of liver biopsies. *Digestive and Liver Disease*.

[9] Bloom S., Kemp W., Lubel J. (2015). Portal hypertension: pathophysiology, diagnosis and management. *Internal Medicine Journal*.

[10] D’Amico G., Garcia-Tsao G., Pagliaro L. (2006). Natural history and prognostic indicators of survival in cirrhosis: a systematic review of 118 studies. *Journal of Hepatology*.

[11] Burtin P., Calès P., Oberti F. (1996). Endoscopic ultrasonographic signs of portal hypertension in cirrhosis. *Gastrointestinal Endoscopy*.

[12] European Association for the Study of the Liver, Asociacion Latinoamericana para el Estudio del Higado (2015). EASL-ALEH clinical practice guidelines: non-invasive tests for evaluation of liver disease severity and prognosis. *Journal of Hepatology*.

[13] Stavropoulos S. N., Im G. Y., Jlayer Z. (2012). High yield of same-session EUS-guided liver biopsy by 19-gauge FNA needle in patients undergoing EUS to exclude biliary obstruction. *Gastrointestinal Endoscopy*.

[14] Miller L., Banson F. L., Bazir K. (2003). Risk of esophageal variceal bleeding based on endoscopic ultrasound evaluation of the sum of esophageal variceal cross-sectional surface area. *The American Journal of Gastroenterology*.

[15] Masalaite L., Valantinas J., Stanaitis J. (2015). Endoscopic ultrasound findings predict the recurrence of esophageal varices after endoscopic band ligation: a prospective cohort study. *Scandinavian Journal of Gastroenterology*.

[16] Carneiro F. O. A. A., Retes F. A., Matuguma S. E. (2016). Role of EUS evaluation after endoscopic eradication of esophageal varices with band ligation. *Gastrointestinal Endoscopy*.

[17] Waxman I., Chapman C. (2018). EUS-guided portal vein sampling. *Endoscopic Ultrasound*.

[18] Tien Y. W., Kuo H. C., Ho B. I (2016). A high circulating tumor cell count in portal vein predicts liver metastasis from periampullary or pancreatic cancer: a high portal venous CTC count predicts liver metastases. *Medicine*.

[19] International Agency for Research on Cancer, World Health Organization (2020). *Cancer Today*.

[20] World Health Organization (2016). *Projections of Mortality and Causes of Death*.

[21] Villanueva A. (2019). Hepatocellular carcinoma. *New England Journal of Medicine*.

[22] Wu J. (2017). The changing epidemiology of hepatocellular carcinoma in Asia versus United States and Europe. *Advances in Modern Oncology Research*.

[23] Loho I. M., Hasan I., Lesmana C. R. A., Dewiasty E., Gani R. A. (2016). Hepatocellular carcinoma in a tertiary referral hospital in Indonesia: lack of improvement of one-year survival rates between 1998-1999 and 2013-2014. *Asian Pacific Journal of Cancer Prevention*.

[24] DeWitt J. M., Arain M., Chang K. J. (2021). Interventional endoscopic ultrasound: current status and future directions. *Clinical Gastroenterology and Hepatology*.

[25] Fusaroli P., Lisotti A., Serrani M., Caletti G. (2018). EUS liver assessment using contrast agents and elastography. *Endoscopic Ultrasound*.

[26] Rimbaş M., Di Maurizio L., Rizzatti G., Gasbarrini A., Costamagna G., larghi A. (2018). Endoscopic ultrasound for the hepatologist: a comprehensive review. *Seminars in Liver Disease*.

[27] Claudon M., Dietrich C., Choi B. (2013). Guidelines and good clinical practice recommendations for contrast enhanced ultrasound (CEUS) in the liver-update 2012. *Ultraschall in der Medizin-European Journal of Ultrasound*.

[28] Liu M., Lin M.-X., Lu M.-D. (2015). Comparison of contrast-enhanced ultrasound and contrast-enhanced computed tomography in evaluating the treatment response to transcatheter arterial chemoembolization of hepatocellular carcinoma using modified RECIST. *European Radiology*.

[29] Sandulescu L., Padureanu V., Dumitrescu C. (2012). A pilot study of real time elastography in the differentiation of focal liver lesions. *Current Health Sciences Journal*.

[30] Hsu Y.-C., Chung C.-S., Tseng C.-H. (2009). Delayed endoscopy as a risk factor for in-hospital mortality in cirrhotic patients with acute variceal hemorrhage. *Journal of Gastroenterology and Hepatology*.

[31] Garcia-Tsao G., Sanyal A. J., Grace N. D., Carey W. (2007). Prevention and management of gastroesophageal varices and variceal hemorrhage in cirrhosis. *Hepatology*.

[32] Nagamine N., Ueno N., Tomiyama T. (1998). A pilot study on modified endoscopic variceal ligation using endoscopic ultrasonography with color doppler function. *American Journal of Gastroenterology*.

[33] Andrade de Paulo G., Ardengh J. C., Nakao F. S., Ferrari A. P. (2006). Treatment of esophageal varices: a randomized controlled trial comparing endoscopic sclerotherapy and EUS-guided sclerotherapy of esophageal collateral veins. *Gastrointestinal Endoscopy*.

[34] Kassem M. A., Salama A. Z., Zakaria S. M., Hassaballah M., Hunter S. M. (2000). Endoscopic ultrasonographic study of the azygos vein before and after endoscopic obliteration of esophagogastric varices by injection sclerotherapy. *Endoscopy*.

[35] Schulman A. R., Thompson C. C., Ryou M. (2016). EUS-guided portal pressure measurement using a digital pressure wire with real-time remote display: a novel, minimally invasive technique for direct measurement in an animal model. *Gastrointestinal Endoscopy*.

[36] Schulman A. R., Ryou M., Aihara H. (2017). EUS-guided intrahepatic portosystemic shunt with direct portal pressure measurements: a novel alternative to transjugular intrahepatic portosystemic shunting. *Gastrointestinal Endoscopy*.

[37] Romero-Castro R., Pellicer-Bautista F. J., Jimenez-Saenz M. (2007). EUS-guided injection of cyanoacrylate in perforating feeding veins in gastric varices: results in 5 cases. *Gastrointestinal Endoscopy*.

[38] Saraswat V. A., Verma A. (2012). Gluing gastric varices in 2012: lessons learnt over 25 years. *Journal of Clinical and Experimental Hepatology*.

[39] Levy M. J., Wong Kee Song L. M., Kendrick M. L., Misra S., Gostout C. J. (2008). EUS-guided coil embolization for refractory ectopic variceal bleeding (with videos). *Gastrointestinal Endoscopy*.

[40] Bhat Y. M., Weilert F., Fredrick R. T. (2016). EUS-guided treatment of gastric fundal varices with combined injection of coils and cyanoacrylate glue: a large U. S. experience over 6 years (with video). *Gastrointestinal Endoscopy*.

[41] Wang X., Yu S., Chen X., Duan L. (2019). Endoscopic ultrasound-guided injection of coils and cyanoacrylate glue for the treatment of gastric fundal varices with abnormal shunts: a series of case reports. *Journal of International Medical Research*.

[42] Romero-Castro R., Ellrichmann M., Ortiz-Moyano C. (2013). EUS-guided coil versus cyanoacrylate therapy for the treatment of gastric varices: a multicenter study (with videos). *Gastrointestinal Endoscopy*.

[43] Robles-Medranda C., Oleas R., Valero M. (2020). Endoscopic ultrasonography-guided deployment of embolization coils and cyanoacrylate injection in gastric varices versus coiling alone: a randomized trial. *Endoscopy*.

[44] Ryou M., McCarty T., Bazarbashi A., Hathorn K., Thompson C. (2020). Combination therapy versus monotherapy for EUS-guided management of gastric varices: a systematic review and meta-analysis. *Endoscopic Ultrasound*.

[45] Varadarajulu S., Jhala N. C., Drelichman E. R. (2009). EUS-guided radiofrequency ablation with a prototype electrode array system in an animal model (with video). *Gastrointestinal Endoscopy*.

[46] Yoon W. J., Daglilar E. S., Kamionek M., Mino‐Kenudson M., Brugge W. R. (2016). Evaluation of radiofrequency ablation using a 1‐Fr wire electrode in porcine pancreas, liver, gallbladder, spleen, kidney, stomach, and lymph nodes: a pilot study. *Digestive Endoscopy*.

[47] Chua T., Faigel D. O. (2019). Endoscopic ultrasound-guided ablation of liver tumors. *Gastrointestinal Endoscopy Clinics of North America*.

[48] Jiang T.-A., Deng Z., Tian G., Zhao Q.-Y., Wang W.-L. (2016). Efficacy and safety of endoscopic ultrasonography-guided interventional treatment for refractory malignant left-sided liver tumors: a case series of 26 patients. *Scientific Reports*.

[49] Hu Y.-H., Tuo X.-P., Jin Z.-D., Liu Y., Guo Y., Luo L. (2010). Endoscopic ultrasound (EUS)-guided ethanol injection in hepatic metastatic carcinoma: a case report. *Endoscopy*.

[50] Faigel D. O., Lake D. F., Landreth T. L., Kelman C. C., Marler R. J. (2016). EUS-guided portal injection chemotherapy for treatment of hepatic metastases: feasibility in the acute porcine model. *Gastrointestinal Endoscopy*.

[51] Choi J.-H., Seo D.-W., Park D. H., Lee S. K., Kim M.-H. (2014). Fiducial placement for stereotactic body radiation therapy under only endoscopic ultrasonography guidance in pancreatic and hepatic malignancy: practical feasibility and safety. *Gut and Liver*.

[52] Garg R., Rustagi T. (2017). Endoscopic ultrasound-guided portal venous access:. *Journal of Clinical Gastroenterology*.

[53] Seo D.-W., Park T., Kang H.-J. (2018). Feasibility and safety of EUS-guided selective portal vein embolization with a coil and cyanoacrylate in a live porcine model. *Endoscopic Ultrasound*.

[54] Chin M. (2018). EUS-guided paracentesis and ascitic fluid analysis. *Endoscopic Ultrasound*.

[55] Suzuki R., Irisawa A., Bhutani M. S. (2014). An automated spring-loaded needle for endoscopic ultrasound-guided abdominal paracentesis in cancer patients. *World Journal of Gastrointestinal Endoscopy*.

[56] Lee S., Seo D.-W., Paik W. H. (2014). Ethanol lavage of huge hepatic cysts by using EUS guidance and a percutaneous approach. *Gastrointestinal Endoscopy*.

[57] Zhang H., Shi G., Sun S. (2017). A case of a giant cyst in the left lobe of the liver successfully treated with endoscopic ultrasound-guided fine needle aspiration (with video). *Endoscopic Ultrasound*.

[58] Ogura T., Masuda D., Saori O. (2016). Clinical outcome of endoscopic ultrasound-guided liver abscess drainage using self-expandable covered metallic stent (with video). *Digestive Diseases and Sciences*.

[59] Ogura T., Takagi W., Onda S. (2015). Endoscopic ultrasound-guided drainage of a right liver abscess with a self-expandable metallic stent. *Endoscopy*.

[60] Chin Y. K., Asokkumar R. (2020). Endoscopic ultrasound-guided drainage of difficult-to-access liver abscesses. *SAGE Open Medicine*.

